# A Chapter on Business Method

**Published:** 1919-04-05

**Authors:** 


### Y.M.C.A. ORGANISATION.

### A Chapter on Business Method.

The work of the Y.M.C.A. for the troops is generally, if vaguely, known, and it is well to have the sketch of its activities which Sir Arthur Yapp has given in "The Romance of the Red Triangle," published by Messrs. Hodder and Stoughton. To have become the "universal provider" of the Army in respect of most things except munitions has involved a vast organisation. In its methods, its advertising, its finance, no institutional worker can fail to be interested. The now familiar hut grew out of the discovery "that no tent could weather the gales of Salisbury Plain in winter," The huts themselves have sometimes been used as emergency hospitals, and, if nothing better offered, a cow-house and a pigsty have been adapted to the ordinary work. A brewery in Earl Street was the first building adapted to sleeping accommodation, and the Euston was the first hostel.

The financial side of the work is curious reading. The first war emergency appeal, which was issued with some misgiving, produced the £25,000 asked for in three days; and by August 1918 the war fund had mounted to two and a half millions. "We have," writes Sir Arthur Yapp, "always been short of money, have always had a big overdraft at the bank, and that largely because we have had to finance a huge business concern without capital." The success of the war fund is attributed to "skilful advertising," "personal solicitation," and "Flag Days"; and the full-page advertisements in the daily newspaper are claimed to be "something quite new in religious and social work advertising." At the same time, of course, the receipts taken over the counters of the huts have been enormous. Sir Arthur Yapp defends the profits which have admittedly been made by saying that the profits "mean further extension," and that "every penny of profit has been spent on the maintenance and development of this work," and argues that a very big turnover may mean a very small profit, because "stamps and postal orders are sold for actual cost," paper and envelopes given free, and that, generally speaking, there are no charges for admission to lectures or entertainments. Games, including cricket and football outfits, are distributed freely, and hot drinks are given to the walking wounded. The expense of motor transport, he adds, brings no return, and the maintenance of hostels for the relatives of wounded in France has been "very costly." So, we learn, has the educational programme. If most huts can be made to pay, some cannot. His conclusion of the matter is that "towards the war services the profits made have been equal to a sum of ten shillings for every pound contributed by the public." In fine, as he puts it, "the Y.M.C.A. might, had it so chosen, have feathered its nest during the war," but it preferred to show "a sublime though by no means a reckless disregard of the future." The principle adopted has been that "the Association hut is not a charity so far as its business side is concerned," but "is expected to pay its way so that the subscriptions from the public can be applied to the extension of the work and to the maintenance of centres tnat cannot be self-supporting." It was eventually decided by Lord Derby, when Secretary of State for War, that the Y.M.C.A. should pay 6 per cent. on its gross takings in huts on military ground to regimental funds. This decision, it is interesting to note, reversed that given by Mr. Asquith when he was at the War Office during Lord Kitchener's absence in Gallipoli.

We confine ourselves to noticing the business side of the work, because this has a technical interest for all administrators.

